# Variations in the Use of mHealth Tools: The VA Mobile Health Study

**DOI:** 10.2196/mhealth.3726

**Published:** 2016-07-19

**Authors:** Kathleen L Frisbee

**Affiliations:** ^1^ Connected Health Office of Informatics and Analytics Veterans Health Administration, Department of Veteran Affairs Washington, DC United States

**Keywords:** caregivers, telemedicine, stress (psychological), veterans health

## Abstract

**Background:**

Mobile health (mHealth) technologies exhibit promise for offering patients and their caregivers point-of-need tools for health self-management. This research study involved the dissemination of iPads containing a suite of mHealth apps to family caregivers of veterans who receive care from the Veterans Affairs (VA) Health Administration and have serious physical or mental injuries.

**Objective:**

The goal of the study was to identify factors and characteristics of veterans and their family caregivers that predict the use of mHealth apps.

**Methods:**

Veteran/family caregiver dyads (N=882) enrolled in VA’s Comprehensive Assistance for Family Caregivers program were recruited to participate in an mHealth pilot program. Veterans and caregivers who participated and received an iPad agreed to have their use of the apps monitored and were asked to complete a survey assessing Caregiver Preparedness, Caregiver Traits, and Caregiver Zarit Burden Inventory baseline surveys.

**Results:**

Of the 882 dyads, 94.9% (837/882) of caregivers were women and 95.7% (844/882) of veteran recipients were men. Mean caregiver age was 40 (SD 10.2) years and mean veteran age was 39 (SD 9.15) years, and 39.8% (351/882) lived in rural locations. Most (89%, 788/882) of the caregivers were spouses. Overall, the most frequently used app was Summary of Care, followed by RX Refill, then Journal, Care4Caregivers, VA Pain Coach, and last, VA PTSD Coach. App use was significantly predicted by the caregiver being a spouse, increased caregiver computer skills, a rural living location, lower levels of caregiver preparedness, veteran mental health diagnosis (other than posttraumatic stress disorder), and veteran age.

**Conclusions:**

This mHealth Family Caregiver pilot project effectively establishes the VA’s first patient-facing mHealth apps that are integrated within the VA data system. Use varied considerably, and apps that were most used were those that assisted them in their caregiving responsibilities.

## Introduction

The US health care system is under tremendous pressure to find ways to reduce costs and improve the quality of care. The responsibility for managing health is shifting from health care providers to patients and their families. This shift reflects an overall trend in health care, moving from a provider-centered delivery system to a patient- and family-centered participatory model of care [1]. This places greater emphasis on patients and family members to assist in the provision of health care. A variety of technologies are being developed in the commercial health market to support self-management, but these technologies need to be available at the point of need to be most useful. One specific group of technologies, the mobile health (mHealth) technologies, shows promise for offering patients and their caregivers’ point-of-need tools for the self-management of health. These mHealth technologies are defined as apps that run on mobile devices for the purpose of assisting consumers or health care providers in monitoring health status or improving health outcomes [2]. mHealth also encompasses sensors, phones, or other devices worn on the body or carried that transmit and receive data wirelessly. mHealth is a subset of the larger field of electronic health (eHealth) that involves the information technologies used in health care delivery [3].

mHealth technologies that run on accessible mobile platforms may be able to accelerate the transformation of health care by empowering patients and their families with the tools and information that have historically resided with health care professionals. Studies have been published that involve the use of mHealth technologies to improve access to care, improve communication between patients and providers, assist patients in their disease management, and support disease monitoring [4-7]. However, research into the factors that influence use and acceptance of mHealth technology has not kept pace with the rapid proliferation of mHealth tools [2,7]. The factors influencing mHealth use and acceptance may be similar to the factors driving other consumer-based eHealth technologies, but evaluations of mHealth tools have been limited to small studies where key variations in use have not been assessed [6].

Technology-based interventions designed to support caregivers and their care recipients have been used with mostly positive results. mCARE, a mobile phone‒based secure messaging system designed for veterans, encompasses several assistive components for patient and caregiver self-management [8]. Some of these components were appointment reminders, self-report assessments, health tips, and secure messaging with their provider. More than 90% of users believed that the mCARE system was somewhat or was easy to use [9], demonstrating that this mHealth app was feasible and effective for this population. A randomized trial was conducted to assess the impact of Comprehensive Health Enhancement Support System (CHESS), a Web-based lung cancer information, communication, and coaching system for caregivers on caregiver burden, disruptiveness, and mood [10]. Caregivers randomized to CHESS reported lower burden and negative mood when compared to those in the Internet group, suggesting that eHealth and mHealth interventions similar to CHESS may improve caregivers’ coping skills and, in turn, decrease their perceived burden levels. Tele-Savvy, an Internet-based version of the in-person, evidence-based psychoeducation Savvy Caregiver Program for caregivers of veterans with dementia, used synchronous (teleconferences) and asynchronous components (video modules) to provide program access to caregivers in their homes [11]. In an effectiveness trial, caregivers demonstrated moderately high initial levels of burden, anxiety, and depressive symptoms, all of which decreased significantly at follow-up. There were slightly significant increases in caregiver competence. While there is notable literature on the positive outcomes associated with already developed eHealth interventions [12], it is critical to continue to understand the needs of the caregiver users.

Numerous studies have shown that in order for technology to be accepted by consumers it must be perceived as beneficial, be easy to use, fit into the workflow of the end user, and be help desk‒supported [13-15]. Understanding what caregivers want from technology-based interventions is important for designing mHealth interventions as well as understanding the factors that will likely drive adoption. Focus groups conducted with community-dwelling patients with complex chronic disease and disability and their caregivers revealed that open two-way communication and dialogue between them and their providers, and better information sharing between providers in order to support continuity and coordination of care as issues that eHealth interventions could address and be of most benefit [16]. Additionally, privacy and data security, accessibility, the loss of necessary visits, increased social isolation, provider burden, shifting responsibility onto patients for care management, entry errors, training requirements, and potentially confusing interfaces were all identified as concerns of patients [16] and therefore need to be taken into consideration when developing eHealth/mHealth technologies. Despite these concerns, upwards of 95% of caregivers who use mobile systems find that interactive features of communication technologies assist in their caregiving [13].

The National Alliance for Caregiving reports that caregivers consistently convey a need for more information including information on keeping the care recipient safe at home (37%), managing their own stress (34%), identifying easy activities to do for their care recipient (34%), and finding time for themselves (32%). Only 24% of caregivers of veterans reported receiving the formal training they need to perform their caregiver responsibilities and a majority feel ill-equipped to deal with the veteran’s condition, both in terms of having confidence in their own skills or knowing how to seek out additional sources of information or support [17]. In a recent survey of 1000 technology-using family caregivers by the National Alliance for Caregiving [18], caregivers were asked to rate 12 technologies on their potential helpfulness to the caregiver. Those technologies that ranked the highest were Personal Health Record Tracking, Medications Support System, and Symptom Monitoring and Transmission. Those technologies rated the lowest were Caregiving Coaching Software, Transportation Display, and Caregiver Mentor Matching Service. The top benefits expected from the technology include saving time, easing the organizational logistics of caregiving, making the care recipient feel safer, increasing the feeling of being effective, and reducing stress. The overriding barrier expected was the expense of the technology, which is echoed in other studies [13].

Using the organizing framework for caregiver interventions devised by Van Houtven et al [19] as a guide, the purpose of this study was to generate new knowledge on the relative rates of use of different mHealth tools and the characteristics of veterans and their family caregivers that would predict their use of mHealth tools. The Caregiver Intervention Organizing Framework has three main directives: (1) interventions should assess the quantity and/or quality of care provided, (2) consider a broader range of caregiver and care recipient outcomes, and (3) consider a common set of caregiver and care recipient outcomes to facilitate comparison across studies and over time [19]. As suggested by the aforementioned framework, the quality of the intervention was assessed by using validated caregiving quality measures, as well as the quantity of care (usage rates). In considering a broader range of caregiver and care recipient outcomes, we assessed several different veteran and caregiver factors that we believed may contribute to use of the intervention. Our caregiver outcomes were measured at several points in time to allow for a longitudinal assessment. The results of this study advance our understanding of the potential for adoption of mHealth tools within the context of caregiving.

## Methods

### Summary

This research study involved the dissemination of iPads (N=881) containing a specific suite of mHealth apps to family caregivers of veterans who receive care in the Veterans Affairs (VA) Health Administration and have serious physical or mental injuries resulting from the post-9/11 wars. Veterans in the study had a combination of physical injuries, mental health diagnoses, and chronic medical conditions, and all were supported by a family caregiver. Thus, these patients exhibit complexities along several axes of the Vector Model of Complexity, a conceptual model that defines patient complexity along axes representing major determinants of health [20]. The suite of mHealth tools was designed by the VA to assist the caregiver in managing veteran posttraumatic stress disorder (PTSD) and pain, as well as provide support with health care-related tasks and help caregivers manage their own stress.

### Study Design and Setting

This study was designed as a prospective cohort study with the objective of better understanding the factors that influence the use of a suite of mHealth tools (apps). The study participants were enrollees in the VA Comprehensive Assistance for Family Caregivers program as of May 2013, who agreed to participate in the VA Family Caregiver Mobile Health Pilot program. The VA Comprehensive Assistance for Family Caregivers program supports the care of post-9/11 veterans and service members who have sustained serious physical or mental injuries because of their service in the military. As part of this program, family caregivers provide personal care services to the eligible veteran in the veteran’s home. The caregivers are eligible to receive a stipend and health insurance if they do not already qualify for it. In addition, the program provides training, counseling, and respite care to support the caregivers in their caregiving role. The Family Caregiver program is staffed by VA Caregiver Support Coordinators who are located at each VA facility and are responsible for making quarterly home visits to families enrolled in the program and provide ongoing support and assistance to these families.

The VA Family Caregiver Mobile Health Pilot is a program that distributed government furnished iPads loaded with VA mHealth tools to VA family caregivers and the veterans they care for. A 1-year data and service plan was provided with the iPads. The mHealth apps were developed by the VA for this mHealth pilot and were available only to pilot participants. This mHealth Family Caregiver Pilot project established the VA’s first patient-facing mHealth apps that are integrated with the VA data system and allowed for the exchange of health-related data between the VA and veterans and their family caregivers.

### Study Population and Recruitment

The study population comprised a cohort of 882 caregiver/veteran dyads that received the iPads, which were loaded with a suite of mHealth apps. A dyad is defined as each caregiver and the unique veteran they provide care for. There were two layers of participation within this study group. The first were caregivers who agreed to participate in the VA mHealth pilot program (N=882). VA administrative data were available for this dyad group, and consent was waived based on its use for secondary data analysis. The second was a subset of caregivers from the study group that completed three baseline surveys (n=577) and consented to participate in this research study. This group will be referred to as the survey group. The Institutional Review Boards of both George Washington University and the Veterans Administration approved the study.

**Figure 1:**
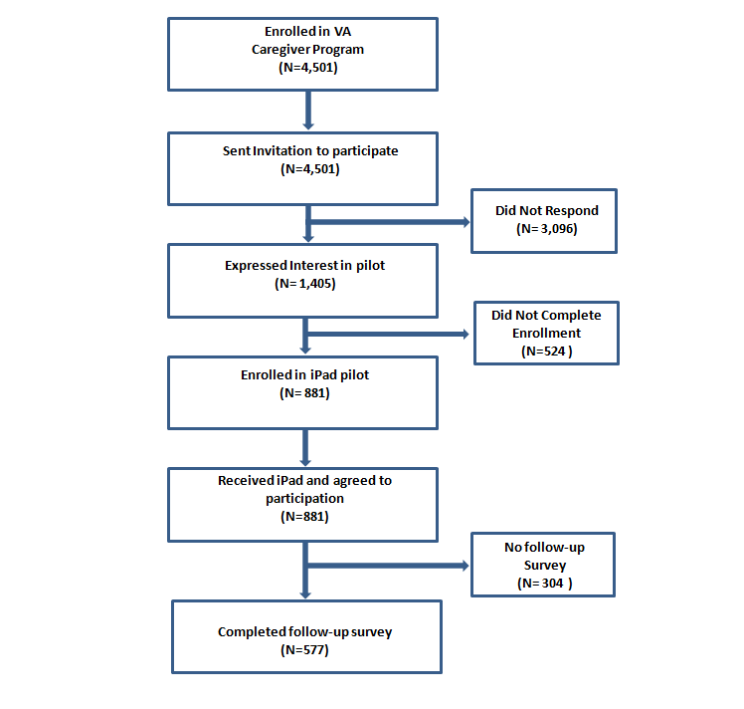
Consort diagram: how the study cohort was formed.

The study group participants were recruited by a letter sent in August 2012 to all 4501 caregivers enrolled in the VA Family Caregiver program, inviting them to participate in the VA Family Caregiver Mobile Health Pilot program. The VA received 23.22% (1045/4501) affirmative responses. Prior to distributing the iPads, caregivers were eliminated for distribution from the original 1045 if they (1) were no longer enrolled in the Family Caregiver program or (2) could not verbally confirm their shipping address. A total of 84.31% (881/1045) of iPads were distributed in late May to June 2013 to caregivers, which represented 882 unique caregiver/veteran dyads (one caregiver had 2 veterans under care, resulting in an additional unique dyad). A second letter was sent to the 881 caregivers in the study group who had agreed to participate in the VA Family Caregiver Mobile Health Pilot program, asking them if they would like to participate in a research study that was intended to help the VA better understand the needs and challenges experienced by those using the mHealth apps. The letter indicated that by completing the initial survey the study participant was giving their consent to participate in the research study. An opt-out postcard was also provided and study participants were asked to return the card if they were not interested in participating in the study. Survey information, from three different surveys, was collected on 65.4% (577/882) of study participants (see Figure 1). The surveys completed by this survey group included the Caregiver Preparedness, Caregiver Traits, and Caregiver Zarit Burden Inventory surveys, which are provided in Multimedia Appendix 1.

### Intervention

The intervention consisted of supplying an iPad loaded with a suite of mHealth apps designed to support caregivers in their caregiving role. Support was provided to users in the form of a quick start guide for setting up the iPad, a website with answers to frequently asked questions, a monthly newsletter, and a Help Desk that received call inquiries. All of the caregivers participating in the study were also called early on to facilitate obtaining a DS Logon (the Department of Veteran Affairs’ self-service account) and were referred to the VA Mobile Health Help Desk for additional assistance.

Several family caregivers/veteran focus groups and usability tests were conducted to assist VA in selecting the types of apps that they would develop and in designing the apps provided in the mHealth pilot. The apps were developed as native iOS apps for the iOS 6 operating system.

**Figure 2:**
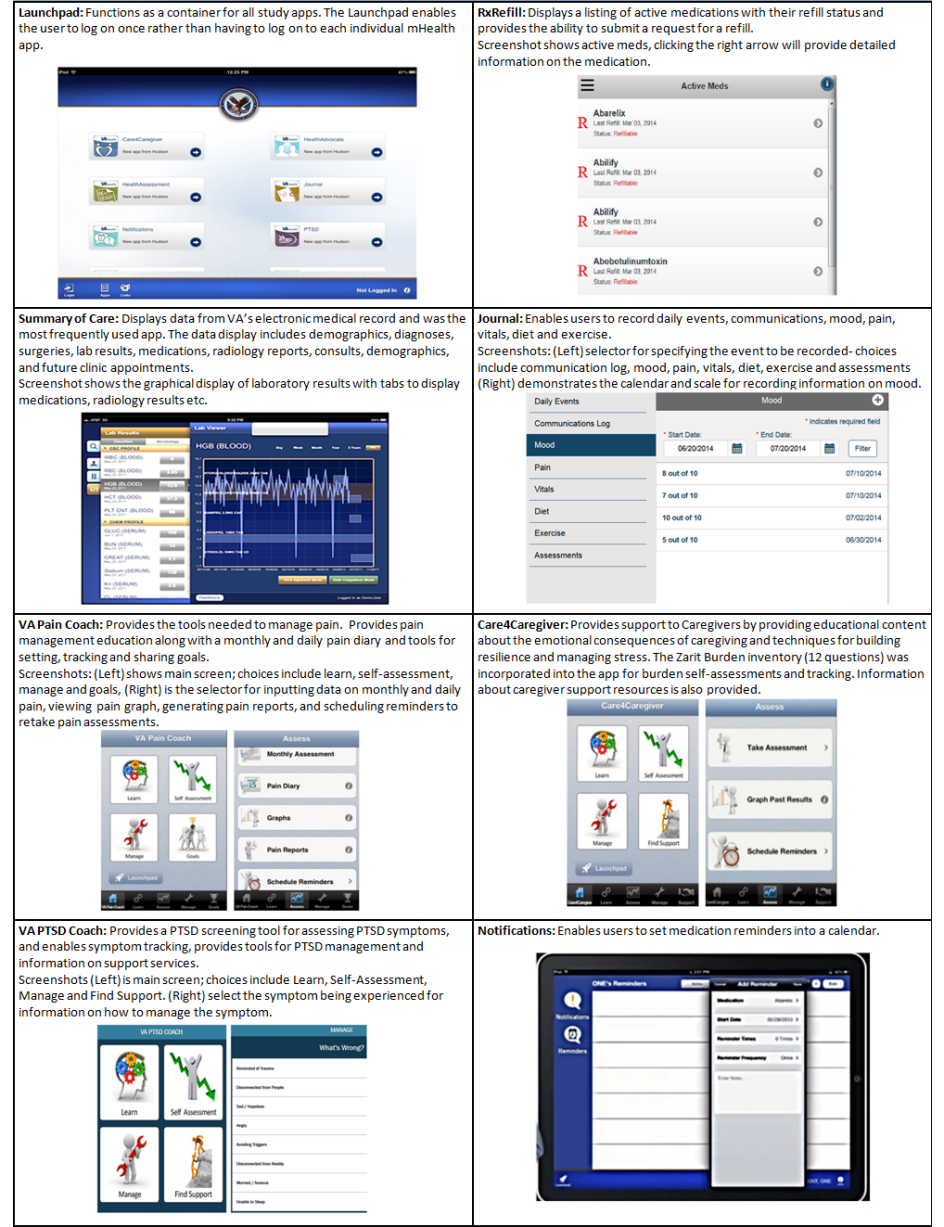
Descriptions and screenshots of the mHealth apps.

The suite of apps was bundled within the Launchpad app, which functioned as the “container” that housed all of the mHealth apps in the study. The Launchpad enabled the user to log on once rather than having to log on to each individual mHealth app. The logon credential used for the mHealth apps was the Department of Defense’s “DS Logon” premium account credential. In many cases, caregivers reported using the veteran’s credentials to log on to the VA mHealth apps instead of their own, thus making it difficult to distinguish whether the caregiver or the veteran was using the app. Figure 2 displays the LaunchPad app and the apps as they appeared within the Launchpad.

### Data Collection

Distribution of the mHealth iPad tools began in late May 2013 and continued through June. Data were collected on the use of these tools for each study participant during their intervention assessment period. The intervention assessment period was defined as the time between when the iPad was received by the study subject and the study end date of September 18, 2013. All the iPads distributed to caregivers were loaded with mobile device management software that allowed the VA to track the use and location of the devices and wipe the devices if they were stolen or manipulated to remove Apple’s security controls. The VA mHealth apps were developed with back-end data metrics that enabled the VA to see the utilization of each VA mHealth app by individual pilot participants and the duration of each use session. Survey data were collected by having study subjects complete three survey instruments that were rendered on the iPads, or by collecting the information verbally over the phone (see Multimedia Appendix 1 for survey items). The survey data fed a back-end database that recorded the date and results of the survey by individual study participant identifier. Descriptive data about the study participants was taken from the VA’s administrative databases. Nonusers of the iPad/mHealth apps intervention were contacted in the early part of the study to determine the reasons for nonuse.

### Study Variables

The study outcome variable was the use of the mHealth/iPad tools. Use was measured in two ways: (1) a binary outcome representing at least one use of the apps versus no use, and (2) the frequency of app use, for those participants using the apps at least once. Frequency of app use was computed as the number of times the app was used during the intervention assessment period. App use was measured for each individual app and for the entire group of apps.

The predictor variables for the study group dyad (N=882) comprised veteran and caregiver characteristics that were obtained from VA administrative databases and which are described in Multimedia Appendix 2. We received a waiver of Health Insurance Portability and Accountability Act authorization to collect this data as it was deemed infeasible to obtain consent for all caregivers enrolled in the VA Caregiver program (N=4501). The predictor variables for the survey group dyad (n=577) consisted of the same administrative predictor variables as the study group dyad and augmented with variables derived from the three self-administered survey instruments. Surveys could be completed on the iPad. If study participants had not completed the surveys on the iPad within 2 weeks of receiving the iPad and had not returned the opt-out postcard, then they were contacted by research staff and were given the opportunity to complete the survey using a telephone interview. The survey instruments are listed in Multimedia Appendix 1.

The caregiver characteristic survey questions represent a subset of questions derived from the 2009 National Alliance for Caregiving survey [17]. These questions include self-reported demographics, activities of daily living, caregiver stress/strain, and computer skills. The caregiver preparedness questions were taken from the Preparedness for Caregiving Scale [21], which asks caregivers to rate themselves on their perceived readiness for the multiple domains of caregiving. The final summary question of the preparedness survey, “Overall, how well prepared do you think you are to care for your Veteran?,” was used as the measure of preparedness because it correlated with the other preparedness questions and had good face validity. The 4-question Zarit Caregiver Burden screening inventory was the survey instrument used to obtain information about caregiver burden levels [22]. The Zarit Caregiver Burden screening inventory is scored with values ranging from 0-4 for each of the four questions. The total possible score is 16. The total score was used as the measure of burden.

### Statistical Analysis

Analysis began by comparing the baseline caregiver/veteran dyad characteristics of the study and survey groups using a chi-square test to determine if the groups differed from one another. Next, a parsimonious set of predictor variables was selected by examining the bivariate relationships between caregiver and veteran dyad characteristics and app use. The strength of the bivariate analysis was assessed, and those variables strongly associated with the outcome variable were reserved as potential predictor variables. Next, a correlation analysis between pairs of potential predictor variables was performed. When two variables were highly correlated, one was dropped or a composite variable was created in order to reduce model multicollinearity. Finally, multivariate modeling was undertaken using SAS version 9.3 software, to predict app use. Logistic regression modeling was performed to predict the binary use/nonuse outcome for the seven apps as a whole. The analysis was then repeated using negative binomial regression modeling. The binary use/nonuse analysis was intended to provide information on the factors associated with initial interest in using the app, while the frequency analysis was intended to provide information on the factors driving sustained use of the app once app use was established.

Multivariate models were assessed for fit. Logistic regression models were evaluated using a Hosmer and Leneshow Goodness of Fit statistic of 0.05 or greater and a C-statistic greater than 0.65. Negative binomial fit was assessed by evaluating if the value of the Pearson chi-square statistic divided by the degrees of freedom was close to the value of 1 and by ensuring that the dispersion parameter was not equal to 0. Model results were assessed using odds ratios in the logistic regression model. Since our models were guided by a specific research purpose, we report each *P* value “as is” without further adjustment for the total number of tests conducted.

## Results

Table 1 displays the characteristics of the study and survey groups. The chi-square analysis of the study group (N=882) and the survey group (n=577) showed that they were not significantly different from one another with respect to their baseline characteristics. In the study group, the majority of caregivers (94.9%, 837/882) were women and the majority of veteran recipients were men (95.7%, 844/882). The average age of the caregiver was 40 years, and the average age of the veteran was 39 years. The caregivers were primarily spouses (89.3%, 788/882) and were geographically dispersed across the United States with 60.0% (529/882) living in urban locations and 39.8% (351/882) living in rural locations.

**Table 1:** Baseline characteristics of caregivers and veterans dyads (N=882).

Baseline characteristics			Study group (N=882)	Survey group (n=577)
**Caregiver characteristics**				
	**Caregiver gender, n (%)**			
		Female	837 (95.01)	547 (96.13)
	Caregiver age, mean (SD)		40.16 (10.20)	40.08 (9.92)
	**Relationship of caregiver to veteran, n (%)**			
		Spouse	788 (89.34)	521 (91.40)
		Parent	69 (7.82)	37 (6.49)
	**Tier funding level for caregiver, n (%)**			
		Tier 1	145 (16.57)	98 (17.28)
		Tier 2	308 (35.20)	199 (35.10)
		Tier 3	422 (48.23)	270 (47.62)
**Veteran characteristics**				
	**Veteran gender, n (%)**			
		Female	38 (4.31)	16 (2.77)
		Male	844 (95.69)	554 (97.19)
	**Veteran race, n (%)**			
		African American	153 (17.35)	88 (15.44)
		Missing	41 (4.65)	25 (4.39)
		Other	54 (6.12)	29 (5.09)
		White	634 (71.88)	428 (75.09)
	**Veteran age group in years, n (%)**			
		≤34	317 (35.94)	208 (36.49)
		≥35 and ≤44	307 (34.81)	193 (33.86)
		≥45 and ≤54	199 (22.56)	134 (23.51)
		≥55	59 (6.69)	35 (6.14)
	**Veteran service-connected category, n (%)**			
		<80	131 (14.94)	86 (15.14)
		≥80	746 (85.06)	482 (84.86)
**Veteran Aid and Attendance recipient, n (%)**			
		No	822 (93.30)	530 (93.15)
		Yes	59 (6.70)	39 (6.86)
	Time in program (days), mean (SD)		529.02 (117.03)	529.00 (117.54)
	Veteran income (US $), mean (SD)		35,038.15 (17,286.83)	35,377.63 (17,316.16)
	Monthly stipend amount (US $), mean (SD)		1534.27 (625.39)	1530.50 (629.94)
	**Veteran marital status, n (%)**			
		Married	766 (87.14)	515 (89.35)
		Divorced	36 (4.10)	17 (2.95)
		Other	77 (8.76)	42 (7.28)
	**Branch of service, n (%)**			
		Army	650 (77.66)	428 (79.11)
		Marines	90 (10.75)	62 (11.46)
		Navy/Coast Guard	57 (6.81)	28 (5.18)
		Air Force	40 (4.78)	23 (4.25)
	**Living location, n (%)**			
		Urban	529 (60.11)	337 (59.23)
		Rural	351 (39.87)	232 (40.77)
	**Veteran diagnoses, n (%)**			
		PTSD	600 (68.03)	390 (68.42)
		Other injury (nerve, multiple fractures)	381 (43.20)	247 (42.33)
		Traumatic brain injury (TBI)	268 (30.39)	180 (31.58)
		Other mental health diagnosis	192 (21.77)	126 (22.11)
		Other illness (diabetes, chronic obstructive pulmonary disease, stroke, cancer)	148 (16.78)	89 (15.61)
		Receiving polytrauma care	144 (16.35)	104 (18.28)
		Spinal cord disorder	77 (8.73)	54 (9.47)
		Amputation	28 (3.17)	18 (3.16)
		Vision impairment	25 (2.83)	13 (2.28)
		Substance abuse	9 (1.02)	7 (1.23)
	**Veteran service utilization, mean (SD)**			
		Mental health visits	3.49 (7.57)	3.20 (6.50)
		Ancillary outpatient visits	4.97 (5.66)	4.91 (5.78)
		Medical outpatient visits	1.92 (2.19)	1.90 (2.16)
		Specialty outpatient visits	0.40 (1.55)	0.42 (1.63)
		Surgical outpatient visits	0.72 (1.50)	0.72 (1.51)
		Other outpatient visits	0.02 (0.20)	0.02 (0.19)

Table 2 displays the outcome variable, App Use, as both distinct users (used at least once) and as frequency of use in the study and survey groups. Table 2 shows that 29.7% (262/882) of the study group never used one of the seven mHealth apps. In the survey group (n=577), the number of nonusers was 13.5% (78/577). An analysis of these nonusers was conducted to understand how many of the caregiver/veterans dyads lacked the DS Logon credentials required to access the VA mHealth Apps. In the study group, 43.1% of the nonusers (113/262) did not have a DS Logon credential and 33% of the nonusers (23/78) in the survey group did not have a DS Logon credential.

**Table 2:** mHealth app use in study and survey groups (N=882).

App name	Study group (=882)				Survey group (n=577)			
	Distinct users	Percentage using	Frequency using	Number of uses per dyad, mean (SD)	Distinct users	Percentage using	Frequency using	Number of uses per dyad, mean (SD)
All Apps	620	0.70	8,794	14.18 (14.22)	499	0.86	7.839	15.71 (14.79)
Notifications	523	0.59	1,652	3.16 (2.71)	429	0.74	1,478	3.45 (2.84)
Summary of Care	522	0.59	2,727	5.22 (5.48)	436	0.76	2.397	5.50 (5.46)
Rx Refill	504	0.57	1,737	3.45 (3.00)	422	0.73	1,522	3.61 (3.01)
Care4Caregivers	372	0.42	756	2.03 (1.40)	327	0.57	690	2.11 (1.43)
Journal	290	0.33	1,102	3.80 (6.58)	244	0.42	988	4.05 (6.99)
VA PTSD Coach	220	0.25	392	1.78 (1.41)	200	0.35	369	1.85 (1.46)
VA Pain Coach	215	0.24	428	1.99 (2.47)	193	0.33	395	2.05 (2.58)

A subset of nonuser caregivers (n=96) were contacted by phone in the early phase of the study to understand the reasons for nonuse of the apps. Main reasons for nonuse included having DS Logon issues (55%, 53/96), having issues with the apps (22%, 21/96), or experiencing other usability issues (9%, 9/96).

**Figure 3:**
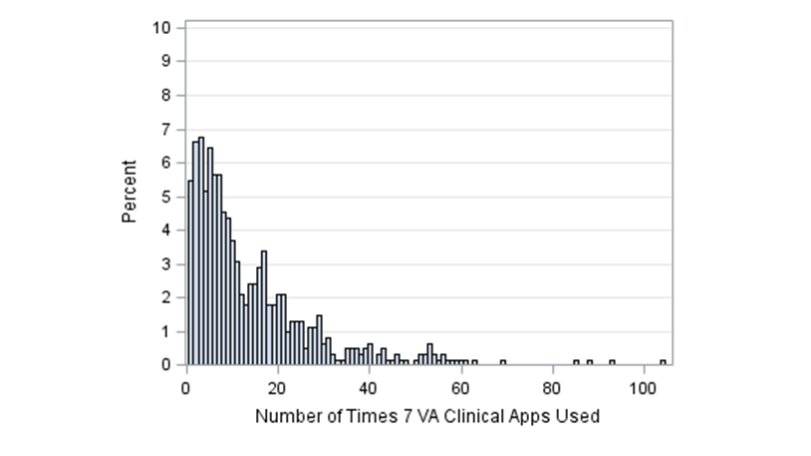
The frequency distribution of total mHealth app use of the caregivers who completed the baseline surveys.

The distribution of the frequency of app use displayed a negative binomial with a zero inflated dispersion. Figure 3 displays the frequency distribution of app use for the seven mHealth Apps as a whole in the survey group (n=577).

The results of the bivariate analysis that crossed each potential predictor variable in the survey group (n=577) with the outcome variable, frequency of mHealth app use, are displayed in Tables 3,4, and 5. Tables 3 and 4 contain caregiver-specific variables and Table 5 contains veteran-specific variables. mHealth app use was categorized into four levels: high (>18 uses), medium (>7 and ≤18), low (> 0 and ≤7), and no use. Use categories were constructed by selecting use ranges that produced three relatively equal groupings among the app users.

**Table 3:** Results of bivariate analysis of caregiver characteristics and frequency of total app use in the survey group.

		Total	High use (>18), (n=195), %	Medium use (>7 and ≤18), (n=175), %	Low use (>0 and ≤7) (n=129), %	No use (n=78), %
**Caregiver age, years**						
	18-34	196	30.1	32.7	26.5	10.7
	35-49	266	38.7	29.3	20.3	11.7
	≥50	115	28.7	28.7 20.0	22.6	
**Caregiver education**						
	Less than High School / High School Grad / GED / Tech School	144	37.5	25.0	22.2	15.3
	Some college/ College grad/ Grad school/ Grad work	433	32.6	32.1	22.4	12.9
**Caregiver race**						
	White	393	34.1	31.8	22.7	11.5
	African American	76	29.0	27.6	26.3	17.1
	Other	108	36.1	26.9	18.5	18.5
Caregiver gender	Female	556	33.6	30.6	22.5	13.3
**Caregiver health**						
	Excellent	48	22.9	33.3	16.7	27.1
	Very good/ Good	437	33.4	30.0	23.6	13.0
	Fair/ Poor	92	41.3	30.4	19.6	8.7
**Urban/Rural** ^a^						
	Rural	234	33.3	33.8	22.2	10.7
	Urban	342	34.2	27.8	22.5	15.5
Relationship^a^	Spouse	527	35.3	29.8	23.0	12.0
**Tech adoption**						
	Early adopter	160	32.5	35.0	21.3	11.3
	Mid adopter	247	36.0	29.2	23.9	10.9
	Late adopter	170	31.8	27.7	21.2	19.4
**Computer skills** ^a^						
	1 or 2 - Limited	39	33.3	23.1	12.8	30.8
	3	131	35.1	25.2	25.2	14.5
	4	208	30.8	34.1	21.6	13.5
	5	174	39.1	32.8	23.6	4.6

^a^Included in the final set of predictor variables.

The influence of caregiver strain, burden, preparedness, and health was most notable in the bivariate analysis, with a high usage associated with poor health, low preparedness, high burden, and high strain. Caregiver age and education showed an association with high use, with middle-aged and lower-educated caregivers showing higher use. Those with higher reported computer skills tended to be higher users of the apps.

**Table 4:** Results of bivariate analysis of caregiving behaviors and frequency of total app use in the survey group.

		Total	High use (>18) (n=195), %	Medium use (>7 and ≤18) (n=175), %	Low use (>0 and ≤7) (n=129), %	No use (n=78), %
Choice in caregiving	Yes	371	35.6	29.7	21.6	13.2
**Hours spent caregiving**						
	≤20 hours	58	34.5	36.2	20.7	8.6
	21-40 hours	139	30.9	32.4	28.8	7.9
	41-80 hours	216	36.1	30.1	20.8	13.0
	≥80 hours	164	32.9	26.8	19.5	20.7
**Caregiver strain**						
	1 - Not a strain at all	128	33.6	29.7	19.5	17.2
	2	198	30.8	28.8	28.8	11.6
	3	184	34.8	33.2	19.7	12.5
	4 or 5 - Very much a strain	67	40.3	28.4	16.4	14.9
**Caregiver stress**						
	1 or 2 - Not at all stressful	186	29.0	28.5	22.6	19.9
	3	165	35.8	29.1	25.5	9.7
	4	134	36.6	32.1	18.7	12.7
	5 - Very stressful	92	35.9	33.7	21.7	8.7
**Years caregiving**						
	≤3 years	237	30.4	29.1	28.3	12.2
	4-7 years	217	39.6	32.3	16.1	12.0
	>7 years	122	30.3	29.5	22.1 18.0	
**Preparedness**						
	Not at all, Not Well, Somewhat Prepared	75	44.0	30.7	16.0	9.3
	Pretty Well Prepared	255	36.9	28.6	25.1	9.4
	Very Well Prepared	228	28.1	32.0	19.7	20.2
**Zarit Burden**						
	High Burden	153	37.9	34.0	20.3	17.9
	Medium Burden	253	34.8	31.2	24.5	9.5
	Low Burden	126	31.0	28.6	16.7	23.8

Similar to caregivers, veterans in the middle-age range were higher users of the apps. Veterans assessed at a monthly stipend level of Tier 1 were higher users. The Tier level represents the amount of work required of the caregiver to meet the care needs of the veteran. Tier 3 represents the highest amount of work and Tier 1 the lowest. Mental health conditions, other than PTSD, were associated with higher app use. Those veterans with a higher percentage of service connected related injuries were associated with lower app use.

**Table 5:** Results of bivariate analysis of veteran characteristics and frequency of app use in the survey group.

		Total	High use (>18) (n=195), %	Medium use (>7 and ≤18) (n=175), %	Low use (>0 and ≤7) (n=129), %	No use (n=78), %
**Veteran age group** ^a^ **, years**						
	≤34	210	28.1	31.4	27.1	13.3
	≥35 and ≤54	332	38.0	30.1	19.3	12.7
	≥55	35	28.6	25.7	22.9	22.9
**Monthly stipend tier**						
	Tier 1	98	40.8	31.6	18.4	9.2
	Tier 2	200 33.0	30.0	25.5	11.5	
	Tier 3	276	31.9	30.1	21.7	16.3
**Veteran race**						
	Black	92	28.3	27.2	26.1	18.5
	Missing	25	32.0	20.0	28.0	20.0
	Other	29	34.5	20.7	34.5	10.3
	White	431	35.0	32.3	20.4	12.3
**Branch of service**						
	Air Force	24	33.3	25.0	25.0	16.7
	Army	430	35.1	31.9	20.9	12.1
	Marines	64	28.1	28.1	26.6	17.2
	Navy/ Coast Guard	30	36.7	26.7	20.0	16.7
TBI DX	1	182	28.0	35.2	19.2	17.6
PTSD DX	1	392	33.9	30.9	21.2	14.0
Other Mental Health DX^a^	1	130	40.8	23.9	27.7	7.7
Other Medical DX	1	97	35.1	29.9	21.7 13.4	
Major Trauma DX	1	69	33.3	18.8	31.9	15.9
Other Nerve Injury DX	1	251	34.3	28.3	21.9	15.5
**Veteran income, US $**						
	<20,000	104	33.7	25.0	26.9	14.4
	20,000-40,000	318	35.2	32.4	21.1	11.3
	>40,000	151	30.5	29.8	21.9	17.89
**Time in program**						
>300 and ≤400	109	37.6	28.4	19.3	14.7	
	>400 and ≤500	145	30.3	30.3	25.5	13.8
	>500 and ≤600	129	32.6	35.7	20.2	11.6
	>600 and ≤700	153	34.6	27.5	23.5	14.4
	>700 and ≤800	41	36.6	29.3	22.0	12.2
**Service connection**						
	<80	86	40.7	27.9	19.8	11.6
	≥80	489	32.7	30.9 22.5	13.9	
Aid and Attendance recipient	Yes	39	25.6	35.9	20.5	18.0
**Marital status**						
	Divorced	17	5.9	35.3	29.4	29.4
	Married	515	34.8	30.9	22.7	11.7
	Other	42	35.7	21.4	16.7	26.2
Polytrauma care^a^	Yes	104	24.0	34.6	20.2	21.2

^a^Included in the final set of predictor variables.

A correlation analysis was performed on the set of potential predictor variables that had a strong association with the outcome variable. Many variables were strongly correlated with one another, for example, caregiver age and veteran age, relationship and marital status, as well as caregiver stress, burden, health, and preparedness, and education with computer skills. A parsimonious set of predictor variables was selected based on the results of the bivariate and correlation analyses. The final set of variables selected for modeling included veteran age, caregiver-veteran relationship, urban-rural living location, other mental health diagnosis, receiving polytrauma care, overall preparedness survey question, and computer skills.

Logistic regression modeling was performed to predict at least one use of the mHealth Apps. Table 6 displays the results of modeling the administrative explanatory for the study group (N=882) (Model 1a) and the administrative explanatory variables plus two additional survey variables, Caregiver Preparedness and Computer Skills, on the survey group (n=577) (Model 2b). The negative binomial analysis demonstrated similar associations (data not shown).

**Table 6:** Logistic regression model predicting at least one use of a clinical mHealth app.

Parameter		Level	Model 1a (N=882)			Model 2b (n=577)		
		OR	95% CI	Pr>χ²	OR	95% CI	Pr>χ²
Assessment period		1.036	1.023-1.050	<.001	1.010	0.975-1.047	.57
Veteran age		0.978	0.962-0.994	.007^c^	0.983	0.953-1.014	.28
Caregiver-veteran relationship	Spouse vs Other	2.428	1.517-3.885	.001^c^	1.591	0.637-3.975	.32
Urban / Rural living location	Rural vs Urban	1.514	1.104-2.075	0.01^c^	1.374	0.777-2.430	.28
Other mental health diagnosis	1 vs 0	1.629	1.104-2.404	0.01^c^	2.602	1.117-6.058	.03^c^
Receiving polytrauma care	No vs Yes	1.260	0.847-1.874	0.25	1.844	0.981-3.468	.06
Computer skills		—	—	—	1.680	1.288-2.191	<.001^c^
Preparedness		—	—	—	0.588	0.381-0.906	.02^c^

^a^Model 1 is based on data from all veteran and caregiver dyads that received the iPad and uses Hosmer and Lemeshow Goodness-of-Fit Test χ^2^=12.76 PR>χ^2^=.121, C-Statistic=0.65.

^b^Model 2 includes all the data from Model 1 and additional survey variables and uses Hosmer and Lemeshow Goodness-of-Fit Test χ^2^=.21 PR>χ^2^=.21 C-Statistic=0.72.

^c^Statistically significant at the .05% level.

For the study group (N=882), significant predictors of using an mHealth App at least once included (1) assessment period—the longer the caregiver/veteran dyad had the mHealth/iPad intervention, the more likely it was they would use it, (2) veteran age—for every one unit increase in age, the likelihood of using a clinical app declined by 0.02%, (3) if the caregiver of the veteran is a spouse, then the odds of using at least one clinical app was 2.4 times greater, (4) those living in a rural location had a 1.5 times greater chance of using a clinical app than those living in a urban location, and (5) veterans with a mental health diagnosis, other than PTSD, were 1.6 times more likely to use a clinical app. When a dummy variable was added to the model to reflect if the caregiver/veteran dyad had a logon credential, veteran age was no longer significant. The significance of this is that veteran age is likely a proxy for the likelihood of having a DS Logon credential. Younger veterans tend to have DS Logon credentials issued when they separated from the service, while this was not true for older veterans.

For survey group (n=577), the only administrative predictor that remained significant was the diagnosis of “Other Mental Health,” which resulted in a 12% increase in the likelihood of using a clinical app compared with those who do not have this diagnosis. Two survey variables were significant predictors. Each one unit increase in caregiver computer skill competency increased the likelihood of using a clinical app by 28%, and each one unit increase in caregiver preparedness decreased the chances of using a clinical app by 42%.

## Discussion

### Principal Considerations

To the author’s knowledge, this is the first study that has looked at factors that predict the use of mHealth apps in the context of caregiving. The study provided a number of key insights. It was found that the mHealth apps used most frequently in this population of caregivers of seriously injured veterans were the Summary of Care, Rx Refill, and Notification apps. Apps used less frequently included the Care4Caregiver Journal, PTSD Coach, and Pain Coach apps. The implication of this finding, based on IT acceptance models, is that use is driven by the perceived usefulness of the app and ease of use [23-25].

The picture that emerged from the bivariate analysis is that there are four principal components driving mHealth app usage. The first relates to the amount of time and effort required for the caregiver to manage the veteran’s medical condition. The second relates to the caregiver strain and preparedness for caregiving. The third has to do with the demographics of the caregivers and veterans. The fourth has to do with computer skills and technology adoption. Caregivers providing care for seriously injured veterans, such as those in polytrauma care or with a high percentage of service-connected conditions as reflected by a high Tier rating (ie, Tier 3) in the caregiver stipend, was associated with decreased app use. This may be related to fewer hours available by the caregiver to use the apps, or it could reflect that use of the apps was a combination of caregiver and veteran use and seriously injured veterans were not as likely to use the apps. The variable selected to represent this dimension in this study was Polytrauma Care. The second component is related to the caregiver’s and veteran care recipient’s physical and mental health condition. Lower health and caregiver preparedness scores coupled with higher strain scores were associated with higher app use. The variable selected to represent the state of the caregiver is Overall Preparedness for Caregiving. The veteran’s medical condition was also an important factor with a diagnosis of a mental health condition, excluding PTSD, being associated with higher usage. Consistent with other studies on factors driving eHealth, demographics were found to be important drivers associated with app use. Increased age of both the veteran and caregiver decreased app use, as did being a non-spouse caregiver. The fourth and final component was related to caregiver computer skills. Those with poor computer skills and low technology adoption rates were less likely to use the Apps; the variable Computer Skills was chosen to represent this dimension.

The results of the logistic regression modeling predicting use versus nonuse of the apps revealed that at least one use of any of the seven study apps was increased by living in a rural location, being a spouse caregiver, being younger, taking care of a veteran with a mental health condition (excluding PTSD), having better computer skills, and feeling less prepared for caregiving. These findings that older individuals and those with lower computer literacy make less use of consumer health technologies is consistent with other research [26,27]. Rural living locations have often been associated with lower eHealth use due to lower Internet access in rural areas [28]. However, in this study, rural living was associated with increased odds of using the mHealth intervention, which is likely associated with the data plans provided to study participants reducing their requirement for Internet access.

The surprising 30% nonuse rate found in the study group deserves further investigation. We know that about 50% of these nonusers did not obtain the proper logon credentials required to use the mHealth intervention. The barriers created by the requirement to obtain user credentials are an important consideration when designing future mHealth apps. Another 30% of nonuse was accounted for by issues the users had with the apps. Although the design of many of the apps was informed by collecting feedback from caregiver focus groups, this finding highlights the need to collect regular feedback from app users to understand usability issues so that these issues can be addressed in subsequent app releases. A surprising finding from this study was the low use of the PTSD app in patients with PTSD. This may be related to the fact that the VA already released a PTSD app to public app stores prior to this study. This was the only one of the study apps that has been released to the public during the course of the study.

### Limitations

It should be noted that this was a pragmatic study examining a target population that is dissimilar to the general patient and caregiver populations, and therefore care must be exercised in extrapolating the results. The study population was restricted to veterans with multiple comorbidities who have sustained serious injuries due to their service in the military. The prevalence of mental health conditions in this population was high and the average age of the population was young, with the average age equal to 39 years. The caregivers in this study were also young, with the average age equal to 40 years old, and therefore do not reflect the typical family caregiver found in the general population. Due to their unique health care needs, future research, both qualitative and quantitative in nature, should aim to evaluate the effects that programs like the Comprehensive Assistance for Family Caregivers program have on veteran/caregiver dyads.

### Conclusions

This study was designed to contribute to our understanding of the factors that drive veteran and caregiver mHealth use within the caregiving context. The mHealth apps that were most used by family caregivers and their veteran recipients were those that provided information from their health care record and assisted them in their caregiving responsibilities, specifically, filling prescriptions and setting medication reminders. This is consistent with previous research indicating that patients value having health information electronically in one place so that it can be shared and used for the management of their health care. Another key finding in this study was that when tablets with data services plans are provided to health care consumers, those in rural areas were more likely to use the technology than those in urban locations. Computer skills and age continue to matter in mHealth usage as they have in other consumer health technologies, reinforcing the need to provide age-target support to avoid disenfranchising older, less computer-savvy individuals. A final key finding of this study was that those caregivers reporting that they are less prepared for caregiving were more likely to use mHealth tools to support their caregiving responsibilities. This mHealth family caregiver VA pilot project was the first to identify predictors of the use of patient-facing mHealth apps that are integrated within the VA data system and that facilitate the exchange of health-related data between the VA and veterans and their family caregivers.

## Appendix

Multimedia Appendix 1 Survey instruments used for baseline data collection.

Multimedia Appendix 2 Description of variables obtained from VA administrative databases.

## Abbreviations

CHESS: Comprehensive Health Enhancement Support System
PTSD: post-traumatic stress disorder
TBI: traumatic brain injury
VA: Veterans Affairs Health Administration
