# Supplementary material for: Variations in the Use of mHealth Tools: The VA Mobile Health Study
Source: JMIR Mhealth Uhealth. 2016 Jul 19;4(3):e89. doi: 10.2196/mhealth.3726 (PMC4971391; doi:10.2196/mhealth.3726)
Supplement: Multimedia Appendix 1 [file mhealth_v4i3e89_app1.pdf]

Appendix 1- Survey Instruments

Baseline Caregiver Characteristics Survey

1. How old are you?
    a. Younger than 18
    b. 18 to 34
    c. 35 to 49
    d. 50 to 64
    e. 65 to 75
    f. 75 or older
2. What is the last grade of school you completed?
    a. Less than high school
    b. High school grad/GED
    c. Some college
    d. Technical school
    e. College grad
    f. Graduate school/Grad work
3. Are you (Check all that apply)
    a. White
    b. African American
    c. Hispanic
    d. Asian
    e. Other
4. Are you
    a. Male
    b. Female
5. Do you feel you had a choice in taking on this caregiving responsibility?
    a. Yes
    b. No
6. Which of the following kinds of help, if any have you provided within the last 21 months to the person you are caring for
    a. Bathing
    b. Getting Dressed
    c. Feeding
    d. Getting in and out of beds or chairs
    e. Help with toileting
    f. Dealing with incontinence or diapers
7. Which of the following kinds of help, if any, have you provided within the last 12 months to the person you are caring for?
    a. Giving medicines, pills or injections
    b. Managing finances (such as bills or insurance paperwork)
    c. Grocery shopping
    d. Housework (such as dishes, laundry, or straightening up)
    e. Preparing meals
    f. Transportation (driving, helping arrange transportation)
    g. Arranging or supervising services

8. Thinking about all of the kinds of help you provide for the veteran you are caring for, how many house do you spend in an average week providing care to him/her?
    a. 8 hours or less
    b. 9 to 20 hours
    c. 21 to 40 hours
    d. 41 to 80 hours
    e. More than 80 hours
9. How would you describe your own health?
    a. Excellent
    b. Very Good
    c. Good
    d. Fair
    e. Poor
10. Think of a scale from 1 to 5, where 1 is not a strain and 5 is very much a strain. How much of a physical strain would you say that caring for your Veteran is for you?
    a. 1 (Not a Strain at All)
    b. 2
    c. 3
    d. 4
    e. 5 (Very Much a Strain)
11. Use the same scale from 1 to 5 where 1 is not at all stressful and 5 is very stressful, how emotionally stressful would you say that caring for your Veteran is for you?
    a. 1 (Not at all Stressful)
    b. 2
    c. 3
    d. 4
    e. 5 (Very Stressful)
12. Which of the following statements best describes your use and adoption of new technology?
    a. You tend to try new technologies when they are relatively new, before most others
    b. You wait a little to see that new technologies are tested, but adopt them more quickly than the average person
    c. You tend to wait until a technology is widely used before you try it
    d. When it comes to adopting new technologies you tend to be one of the very last to try something new
13. Use the same scale from 1 to 5 where 1 is having limited computer skills and 5 is having excellent computer skills, rate yourself on your computer skills and capabilities
    a. 1 (Limited computer skills)
    b. 2
    c. 3
    d. 4
    e. 5 (Excellent computer skills)
14. Indicate the number of years that you have been in the role of family caregiver for your Veteran?
    a. Less than 1 year
    b. 1 to 3 years
    c. 4 to 7 years
    d. > 7 years

Zarit Caregiver Burden Instrument (4-Question)

ZARIT BURDEN SURVEY

For each question listed below check the column that reflects how often you feel this way

Question	Never	Rarely	Sometimes	Quite Frequently	Nearly Always
Do you feel that because of the time you spend with your Veteran that you don't have enough time for yourself?					
Do you feel stressed between caring for your Veteran and trying to meet other responsibilities (work/family)?					
Do you feel strained when you are around your Veteran?					
Do you feel uncertain about what to do about your relative?					

Caregiver Preparedness Survey

CAREGIVER PREPAREDNESS SURVEY

For each question listed below check the column that reflects how prepared you feel as a Caregiver.

Question	Scale of Preparedness				
	Not at All Prepared	Not Too Well Prepared	Somewhat Well Prepared	Pretty Well Prepared	Very Well Prepared
How well prepared do you think you are to take care of your Veteran's physical needs					
How well prepared do you think you are to take care of your Veteran's emotional needs?					
How well prepared do you think you are to find out about and set up services for your Veteran?					
How well prepared do you think you are for the stress of caregiving					
How well prepared do you think you are to respond to and handle emergencies that involve your Veteran?					
How well prepared do you think you are to get the help and information you need from the VA's Health Care System?					
Overall, how well prepared do you think you are to care for your Veteran?					
