# Supplementary material for: Variations in the Use of mHealth Tools: The VA Mobile Health Study
Source: JMIR Mhealth Uhealth. 2016 Jul 19;4(3):e89. doi: 10.2196/mhealth.3726 (PMC4971391; doi:10.2196/mhealth.3726)
Supplement: Multimedia Appendix 2 [file mhealth_v4i3e89_app2.pdf]

Appendix 1 - Description of Variables Obtained from VA Administrative Databases

**Caregiver and Veteran Characteristics Obtained from VA Administrative Databases**	
**Variable**	**Description**
Veteran Marital Status*	Marital Status
Veteran Race	Race collapsed to White / African American / Other
Veteran Age*	Veteran Age as of June 1, 2013
Veteran Income	Veteran Reported Income
Veteran Branch of Service*	Branch of Service Cost Guard collapsed with Navy
Caregiver Age*	Caregiver Age as of June 1, 2013
Caregiver Gender	Caregiver Gender
Relationship*	Relationship of Caregiver to Veteran
Urban / Rural Living Location	Urban Rural Location
Diagnosis TBI	Traumatic Brain Injury Diagnosis in
Diagnosis PTSD	Post-Traumatic Stress Disorder Diagnosis
Diagnosis Other Mental Disorders	Mental Disorders excluding PTSD (e.g. Depression, Bipolar, Schizophrenia)
Diagnosis Other Medical Illness	Other Medical Diagnoses such as Diabetes, COPD, Arthritis, Stroke, Cancer
Diagnosis Amputation	Amputation Diagnosis
Diagnosis Spinal Injury Disorder	Spinal Cord Injury or Disorder Diagnosis
Diagnosis Vision Impairment	Vision Impairment Diagnosis
Diagnosis Other Injury (e.g. nerve, fracture)	Other nerve injury or multiple fractures
Diagnosis Substance Abuse	Substance Abuse diagnosis
AA Recipient*	Vet Receiving Aid and Attendance Assistance
Outpatient Visits to Ancillary Clinics *	Outpatient Ancillary visits computed for 6 months prior to study
Outpatient Visits to Medicine Clinics *	Outpatient Medicine visits computed for 6 months prior to study
Outpatient Visits to Surgical Clinics	Outpatient Surgical visits computed for 6 months prior to study
Outpatient Visits to Mental Health Clinics	Outpatient Mental Health visits computed for 6 months prior to study
Outpatient Visits to Specialty Clinics	Outpatient Specialty Care visits computed for 6 months prior to study
Outpatient Visits to Other Clinics	Outpatient Ancillary visits computed for 6 months prior to study
Service Connection	Veterans Service Connection Rating Category
Veteran receiving Polytrauma Care	Receiving Poly Trauma Care
Time in Program*	Length of Time Enrolled in Family Caregiver Program
Monthly Stipend Amount	Amount paid to Caregiver by VA for performing Caregiving duties
Tier	Tier 1 - lowest; assessed by VA based on caregiving responsibilities
